# Rehabilitation and Biomarkers of Stroke Recovery: Study Protocol for a Randomized Controlled Trial

**DOI:** 10.3389/fneur.2020.618200

**Published:** 2021-01-15

**Authors:** Alessandro Picelli, Mirko Filippetti, Lidia Del Piccolo, Federico Schena, Leonardo Chelazzi, Chiara Della Libera, Massimo Donadelli, Valeria Donisi, Paolo Francesco Fabene, Stefania Fochi, Cristina Fonte, Marialuisa Gandolfi, Macarena Gomez-Lira, Elena Locatelli, Giovanni Malerba, Sofia Mariotto, Chiara Milanese, Cristina Patuzzo, Maria Grazia Romanelli, Andrea Sbarbati, Stefano Tamburin, Massimo Venturelli, Paola Zamparo, Alessandra Carcereri de Prati, Elena Butturini, Valentina Varalta, Nicola Smania

**Affiliations:** Department of Neurosciences, Biomedicine and Movement Sciences, University of Verona, Verona, Italy

**Keywords:** clinical neurophysiology, cognition, microbiota, microRNAs, movement, oxidative stress, psychology, prognosis

## Abstract

**Background:** Stroke is a leading cause of disability. Nonetheless, the care pathway for stroke rehabilitation takes partially into account the needs of chronic patients. This is due in part to the lack of evidence about the mechanisms of recovery after stroke, together with the poor knowledge of related and influencing factors. Here we report on the study protocol “Rehabilitation and Biomarkers of Stroke Recovery,” which consists of 7 work-packages and mainly aim to investigate the effects of long-term neurorehabilitation on stroke patients and to define a related profile of (clinical-biological, imaging, neurophysiological, and genetic-molecular) biomarkers of long-term recovery after stroke. The work-package 1 will represent the main part of this protocol and aims to compare the long-term effects of intensive self-rehabilitation vs. usual (rehabilitation) care for stroke.

**Methods:** We planned to include a total of 134 adult subacute stroke patients (no more than 3 months since onset) suffering from multidomain disability as a consequence of first-ever unilateral ischemic stroke. Eligible participants will be randomly assigned to one of the following groups: intensive self-rehabilitation (based on the principles of “Guided Self-Rehabilitation Contract”) vs. usual care (routine practice). Treatment will last 1 year, and patients will be evaluated every 3 months according to their clinical presentation. The following outcomes will be considered in the main work-package: Fugl-Meyer assessment, Cognitive Oxford Screen Barthel Index, structural and functional neuroimaging, cortical excitability, and motor and somatosensory evoked potentials.

**Discussion:** This trial will deal with the effects of an intensive self-management rehabilitation protocol and a related set of biomarkers. It will also investigate the role of training intensity on long-term recovery after stroke. In addition, it will define a set of biomarkers related to post-stroke recovery and neurorehabilitation outcome in order to detect patients with greater potential and define long-term individualized rehabilitation programs.

**Clinical Trial Registration:** www.ClinicalTrials.gov, identifier: NCT04323501.

## Introduction

Stroke is a leading cause of death worldwide with an incidence of 150–300/100,000 per year. Despite the improvements in prevention and treatment of the acute phase, stroke remains the main cause of adult disability in Western Countries, with 40% of stroke survivors reporting problems in activities of daily living (1, 2). Currently, in Italy there are over one million people suffering from long-term disability after stroke (3). Nonetheless, the care pathway for stroke rehabilitation focuses mainly on the first period (a few weeks or a few months) after acute stroke, taking partially into account the needs of patients during the (short- and long-term) chronic phase. This is further stressed by the difficult access to rehabilitation and care after discharge. In our view this organizational model is also due to the lack of evidence about the mechanisms of recovery in the subacute and chronic phases of stroke together with the poor knowledge of related and influencing factors (3). Along these lines, within the framework of multidomain disability (sensorimotor, cognitive-behavioral, autonomic, and psychosocial) affecting patients with stroke it is necessary to define a number of clinical, functional, neurophysiological, neuroimaging, genetic-molecular, and psychological factors that correlate prospectively with long-term recovery and disability after stroke. This is in order to describe a profile of biomarkers that characterizes stroke patients with greater recovery potential, on which to base the development of innovative treatment protocols for long-term rehabilitation (4–8).

Here, we report the protocol of the study “Rehabilitation and biomarkers of stroke recovery” (ClinicalTrials.gov identifier NCT04323501), which is a single blind, randomized, controlled (parallel-group) trial consisting of seven work packages (WP). Its aim is to investigate the effects of a treatment protocol based on self-management for long-term neuromotor rehabilitation of stroke patients and to define a related profile of clinical-biological, imaging, neurophysiological, and genetic-molecular biomarkers of long-term recovery after stroke.

WP1 represents the interventional part of this protocol and compares the long-term effects of intensive self-rehabilitation vs. the usual rehabilitation care for patients with chronic stroke. Furthermore, it aims to define a set of neuroimaging (diffusion tensor imaging and functional magnetic resonance imaging) and neurophysiological biomarkers (motor evoked potentials and intracortical excitability measured by single and paired transcranial magnetic stimulation, somatosensory evoked potentials, and brain connectivity measured by electroencephalographic phase synchrony) related to the long-term outcome of neuromotor rehabilitation. WP2 aims to investigate cognitive plasticity mechanisms related to neuromotor recovery after long-term rehabilitation in patients with stroke. WP3 explores fatigue, body composition and walking issues in relation with long-term rehabilitation outcome after stroke. WP4 aims to define some psychological aspects, including distress, pain, and motivation, related to the long-term rehabilitation process and post-stroke outcome. WP5, WP6, and WP7 aim, respectively, to describe some issues about microbiota, microRNA expression, and oxidative stress phenomena associated with the mechanisms of post-stroke recovery in relation to long-term rehabilitation management (9).

## Methods and Materials

The study will be conducted at the Department of Neurosciences, Biomedicine, and Movement of Verona, University in Verona, Italy.

### Participants

As to eligibility for this protocol, the inclusion criteria are the following: age >18 years; multidomain disability as a consequence of first-ever unilateral ischemic stroke documented by computerized tomography scan or magnetic resonance imaging; and no more than 3 months since stroke onset; ability of patient and/or caregiver to understand the instructions provided for self-rehabilitation. Exclusion criteria are the following: inclusion in other trials; Mini Mental State Examination score <24/30 (10); drug addiction; and other neurological or orthopedic conditions limiting the inclusion in this protocol. Further eligibility criteria for WP2 are cognitive disability with adequate comprehension skills (people with limited attention span excluded). As to other additional eligibility criteria, WP3 will include patients with stroke who are able to walk and have adequate comprehension skills once they have reached the chronic phase of illness. An extra eligibility criterion for WP4 is the presence of painful conditions. The study does not include vulnerable populations such as prisoners, legally interdicted persons, or institutionalized people.

### Recruitment

Subjects will be recruited among consecutive patients referring to the Neurorehabilitation Unit of the University Hospital of Verona in Italy. All participants will have to give their informed written consent to participate in the study.

### Randomization, Allocation Concealment, and Blinding

Prior to testing, eligible participants are randomly assigned in a 1:1 ratio to one of the two interventions of WP1: intensive self-rehabilitation (Group 1) vs. usual rehabilitation care (Group 2). The patients will be allocated to one of the two intervention arms according to a balanced (restricted) software-generated randomization scheme. Once the decision has been made, the investigator, who has determined whether a subject is eligible for the trial, is unaware of which group the subject would be allocated to (with the allocation contained in opaque sealed envelopes). Another investigator will check for correct patient allocation according to the randomization list. Investigators who assess outcomes are blinded to the intervention performed.

### Interventions

Both types of interventions will last 1 year.

#### Self-Rehabilitation

Patients allocated to this group will undergo an intensive self-rehabilitation protocol based on the principles of “Guided Self-Rehabilitation Contract” (11, 12). This approach consists of an antagonist-targeting self-rehabilitation treatment supported by a diary and based on a moral contract between patient and therapist. In particular, therapists will define, teach, and readjust over time a daily program of self-stretch postures and training exercises according to the needs and progress of each patient. Furthermore, the therapist will provide each patient with an illustrated manual that describes the training program. The stretching program involves static postures of stretching at a high load below the pain threshold for specific antagonist muscle groups; tension on the stretched muscles should be kept for a cumulative period of ≥10 min a day per targeted muscle. The training program includes a series of unassisted rapid alternating movements (or movement efforts) of possible maximum amplitude against targeted antagonist muscles in a short time (e.g., 15–30 s per series, depending on fatigability). At each visit a diary will be requested from the patient reporting the actual practice of self-treatment (e.g., daily time, number of repetitions).

#### Usual Rehabilitation Care

In this group, physiotherapy sessions will be freely prescribed by the patient's physiatrist, according to the usual care practice, which take into account not only medical opinions but also the patient's needs and requests. This community-based therapy sessions are universally and indefinitely covered by the public National Health System in Italy. Physiotherapists providing rehabilitation sessions in this group will be freely selected by the patient and/or recommended by the physiatrist, as in routine practice.

### Outcomes

The primary endpoint of WP1 is comparison of sensorimotor disability after self-rehabilitation vs. usual care treatment. Accordingly, its primary outcome is Fugl-Meyer assessment (FMA), which comprises five domains (155 items in total) as follows: motor function of the upper and lower extremities; sensation, tested through a light touch on two surfaces of the arm and leg, as well as position sense for eight joints; balance, three seated and four standing tests; joint range of motion, testing eight joints; and joint pain. Items are scored on the basis of ability to complete the item, according to which 0 = cannot perform; 1 = performs partially; and 2 = performs fully. The maximum total scale score is 226. Points are divided among the domains as follows: the motor function score ranges from 0 (hemiplegia) to 100 points (normal motor performance) and is divided into 66 points for the upper extremities and 34 points for the lower extremities; the sensation score ranges from 0 to 24 points and is divided into 8 points for light touch and 16 points for position sense; the balance score ranges from 0 to 14 points and is divided into 6 points for sitting and 8 points for standing; the joint range of motion and pain scores range from 0 to 44 points (13).

The secondary endpoint of WP1 is comparison of cognitive and global disability after self-rehabilitation vs. usual care treatment. Accordingly, its secondary outcomes are the Cognitive Oxford Screen (OCS) and the Barthel Index (BI). The OCS is structured on five cognitive domains (namely, attention and executive function, language, memory, number processing, and praxis) and consists of 12 tasks as follows: picture naming (score range 0–4); semantics (score range 0–3); orientation (score range 0–4); visual field (score range 0–4); sentence reading (score range 0–15); number writing (score range 0–3); calculation (range score 0–4); broken hearts (range score 0–50); imitation (range score 0–12); recall and recognition (range score 0–4 each); and executive task (range score 0–13). The cutoffs are adjusted for age, gender, and years of education (14). The BI measures the extent to which somebody can function independently and has mobility in their activities of daily living (i.e., feeding, bathing, grooming, dressing, bowel control, bladder control, toileting, chair transfer, ambulation, and stair climbing). The score ranges from 0 to 100 (15). Other factors considered in the WP1 will be structural and functional neuroimaging, cortical excitability, motor, and somatosensory evoked potentials.

Evaluation endpoints of WP2 include the ability to process visual-spatial information consciously or unconsciously by means of reaction times and to allocate selective attention to the environment around the patient; the ability to perceive relevant elements for behavioral attitudes by means of eye movements and to transform sensory information into appropriate movements, in both a reactive and a predictive way. Accordingly, the outcomes include behavioral measures—e.g., reaction times, eye movements and limb movements—and instrumental measures—e.g., cortical excitability and cortical connectivity—assessed by means of paired transcranial magnetic stimulation and electroencephalographic phase synchrony.

The evaluation endpoints of WP3 include the measurements of fatigue, body composition, and gait. Accordingly, the outcomes include the Multidimensional Fatigue Inventory, which aims to measure the frequency and severity of global fatigue, with a score ranging from 0 to 100, and to define the type of physical/muscular and mental fatigue (16). Other measures include body densitometry (DEXA), walking energetics, and mechanics.

Evaluation endpoints of WP4 include incidence of psychopathology (e.g., anxiety and depression) after stroke and related pain conditions, psychopathological issues, and coping strategies affecting adherence to rehabilitation programs, effectiveness of psychopharmacological and psychotherapeutic treatments in stroke patients, definition of specific psychological interventions supporting rehabilitation (17, 18). Accordingly, the outcomes considered are the Symptom Checklist-90 scale (19); the Eysenck Personality Questionnaire-R (20); the COPE scale (21); the General Self-Efficacy Scale (22); the Acceptance and Action Questionnaire-II scale (23); the Multidimensional Scale of Perceived Social Support (24); the Brief Pain Inventory-I scale (25).

The main endpoint of WP5 is to evaluate changes in microbiota related to post-stroke condition and recovery. Accordingly, the gut microbiota (GM) composition and changes will be evaluated at different time points. To identify bacterial species, present in GM, analysis of nine variable regions of 16S ribosomal RNA gene will be performed by next generation sequencing (NGS). Bioinformatical and statistical analyses will be conducted to relate gut microbiota signatures at different taxonomic levels to stroke rehabilitation conditions (25).

The main endpoint of WP6 is to evaluate the expression of microRNAs (miRNAs) related to post-stroke condition and recovery. Accordingly, in this study, the expression levels of circulating miRNAs (miR-9; miR-29a- 3p; miR-155-5p; miR-124; miR-146a; miR-210; miR-371-3p; miR-495-3p; miR-941; spike-in) will be analyzed at different time points starting from serum samples.

The main endpoint of WP7 is to evaluate the relationship between stroke, oxidative stress, and neuronal death issues. Accordingly, the following concentrations will be analyzed at different time points starting from plasma samples: glutathione disulphide ratio [GSH]/[GSSG]; nitrite/nitrate ratio [NO2^−^]/[NO3^−^]; and post-translational oxidative modifications of plasma proteins. Furthermore, the activation of STAT1 signaling will be evaluated in Peripheral Blood Mononuclear Cells (PBMC).

Evaluation time points for all WPs are reported in Table 1.

**Table 1:** Evaluation time points.

	**T0°**	**T1°**	**T2°**	**T3°**	**T4°**
	**First week**	**3 months**	**6 months**	**9 months**	**12 months**
Informed consent	✓				
Inclusion and exclusion criteria	✓				
Clinical history	✓				
Fugl-Meyer Assessment	✓	✓	✓	✓	✓
Oxford Cognitive Screen	✓	✓	✓	✓	✓
Barthel Index	✓	✓	✓	✓	✓
Volumetric analysis of the stroke	✓				
Side and site of stroke	✓				
DTI	✓				
fMRI	✓				✓
MEP and cortical excitability to TMS	✓				✓
SSEP	✓				✓
EEG	✓		✓		
MFI	✓		✓		
DEXA			✓		
Microbiota			✓		
Biochemistry and miRNA analysis	✓				✓
Usual care	✓	✓	✓	✓	✓
Intensive self-rehabilitation program	✓	✓	✓	✓	✓
Physiatric evaluation	✓	✓	✓	✓	✓
Video analysis and energetic cost of the gait	✓	✓	✓	✓	✓
Psychological evaluation scales	✓	✓	✓	✓	✓
Informed consent	✓	✓	✓	✓	✓

*DTI, Diffusion Tensor Imaging; fMRI, functional Magnetic Resonance Image; MEP, Motor evoked potential; SEP, Somatosensory evoked potential; TMS, Transcranial Magnetic Stimulation; EEG, Electroencephalography; MFI, Multidimensional Fatigue Inventory; DEXA, Dual X-ray Absorptiometry; miRNA, microRNA. °Time from the enrollment*.

### Sample Size

We estimated that 88 subjects, 44 patients per group, would provide 80% power (α level 5%) to detect a difference of 7 points (SD 11.6) on the FMA, the primary outcome, between the groups (26). Considering a dropout rate of 10%, we planned to enroll a total of 97 patients in this study.

### Data Analysis

Adequate descriptive statistics will be produced, including frequency, mean, median, standard deviation, interquartile range, and confidence intervals, for the demographic data and outcome measures at each time point. As to WP1, the comparison between groups, in terms of self-rehabilitation vs. usual care, with regard to the outcomes considered at each time point of the various evaluation times, will be performed using parametric (e.g., *t*-test, ANOVA) and/or non-parametric statistics, such as the Kruskal-Wallis test or the Mann-Whitney test, depending on the data distribution. Correlation and regression analyses will also be performed between neuroimaging data, neurophysiological variables, and clinical outcomes at different time points. As to the other WPs (2–7), comparisons between groups at different times will be assessed using parametric tests, such as the Student *t*-test and ANOVA, or non-parametric tests, such as the Kruskal-Wallis test and Mann-Whitney test, depending on the data distribution. For each variable considered, both longitudinally and transversely, correlation and regression analyses will also be performed. Moreover, for each WP we will perform longitudinal intragroup comparisons at different times by means of parametric tests, such as the *t*-test and ANOVA and/or non-parametric tests, such as the Friedman test and the Wilcoxon test, depending on data distribution. The statistical analysis will be performed using the Statistical Package for Social Science SPSS® version 26.0 software for Macintosh (SPSS Inc., Chicago, IL, USA). For all statistical tests, the significance level is set at *p* < 0.05.

## Discussion

This trial will evaluate and follow up stroke patients from subacute stroke to the chronic phase, up to 1 year after stroke onset. It will deal with the effects of an intensive self-management rehabilitation protocol and a related set of biomarkers. In our view it will add some important pieces of evidence to overcome current conceptual limitations on long-term recovery models, upon which the usual care pathway of stroke rehabilitation is based. Another aspect that will be investigated by this study is the role of training intensity on long-term recovery after stroke, a point that will be further strengthened by the moral contract between patients and therapists (12). As suggested in the literature, intensity is a key element of stroke rehabilitation in order to achieve greater training effects (2, 27). In addition, a pattern of biomarkers related to post-stroke recovery and neurorehabilitation outcome will be useful to detect those patients with greater potential for recovery. Despite the fact that some of these biomarkers have been previously reported to offer information on stroke rehabilitation outcome, the prognostic relevance of their combination has never been explored to date and may be important for defining future long-term personalized medical rehabilitation programs. The possible main problems concerning the conduct of the study will be the number of patients enrolling, this being a single center study, the long follow-up duration (1 year), and the multifactorial/multimodal approach to evaluate patients; (that is, many different outcomes will be considered).

### Ethics and Dissemination

#### Ethics, Privacy, Data Security, and Safety

This study has been approved (approval number 2320CESC) by the local Ethics Committee (Comitato Etico per la Sperimentazione Clinica delle Province di Verona e Rovigo) and will be carried out according to the Declaration of Helsinki. All data and biological samples collected during the study will be handled fairly. The database will remain strictly confidential, anonymized, password protected, and stored together with biological samples in locked and secured facilities at the Department of Neuroscience, Biomedicine, and Movement Sciences of Verona University. Participants will sign an informed consent form and will be assigned with a unique anonymous identifier. Only authorized study personnel will have access to the match between the identifier and identity of patients. The identifier of each patient will remain the same during the whole study and will allow the proper handling of data and biological samples. Data will be collected in a dedicated case report form and added to an electronic database for analysis.

#### Dissemination Policy

A dissemination plan will be established during the first phase of the study in agreement with all researchers by means of dedicated meetings. The dissemination target will be different stakeholders (e.g., other researchers, representatives and associations of patients, healthcare professionals, and the general population). Each dissemination product will be tailored to these different target groups in terms of objectives, methods, timeline, and language. Websites and social networks will also be used to disseminate the project issues. The use of these social media channels will allow a broader dissemination of the study results. The scientific community will be reached through scientific publications in national and international (preferably open access) journals as well as by means of presentations (keynotes and posters) at national and international congresses. Items for dissemination in any format including publications will not contain information leading to the identification of patients.

## Ethics Statement

This study involving human participants was reviewed and approved by Comitato Etico per la Sperimentazione Clinica delle Province di Verona e Rovigo. The patients provided their written informed consent to participate in this study.

## Author Contributions

AP, MF, and NS wrote the initial draft of the manuscript. All authors contributed to revision and final approval of the manuscript.

## Conflict of Interest

The authors declare that the research was conducted in the absence of any commercial or financial relationships that could be construed as a potential conflict of interest.
